# SEOM-GEMCAD-TTD clinical guideline for the diagnosis and treatment of gastric cancer (2023)

**DOI:** 10.1007/s12094-024-03600-7

**Published:** 2024-07-18

**Authors:** Fernando Rivera, Federico Longo, Marta Martín Richard, Paula Richart, Maria Alsina, Alberto Carmona, Ana Belén Custodio, Ana Fernández Montes, Javier Gallego, Tania Fleitas Kanonnikoff

**Affiliations:** 1 Medical Oncology Department, Hospital Universitario Marqués de Valdecilla, IDIVAL, Santander, Spain; 2 Medical Oncology Department, Hospital Universitario Ramón y Cajal, Madrid, Spain; 3 Medical Oncology Department, ICO Duran i Reynals, Barcelona, Spain; 4 Medical Oncology Department, Hospital La Fe, Valencia, Spain; 5 Medical Oncology Department, Hospital Universitario de Navarra (HUN), Pamplona, Spain; 6 Medical Oncology Department, Hospital Morales Meseguer, Murcia, Spain; 7 Medical Oncology Department, Hospital Universitario La Paz, Madrid, Spain; 8 Medical Oncology Department, Complexo Hospitalario Universitario de Ourense (CHUO), Ourense, Spain; 9 Medical Oncology Department, Hospital General Universitario de Elche, Alicante, Spain; 10 Medical Oncology Department, Hospital Clínico Universitario de Valencia, INCLIVA, Valencia, Spain

**Keywords:** Gastric cancer, Tumor biology, Precision medicine, Immunotherapy, Supportive care

## Abstract

Gastric cancer (GC) is the fifth most common cancer worldwide with a varied geographic distribution and an aggressive behavior. In Spain, the incidence is lower and GC represents the tenth most frequent tumor and the seventh cause of cancer mortality. Molecular biology knowledge allowed to better profile patients for a personalized therapeutic approach. In the localized setting, the multidisciplinary team discussion is fundamental for planning the therapeutic approach. Endoscopic resection in very early stage, perioperative chemotherapy in locally advanced tumors, and chemoradiation + surgery + adjuvant immunotherapy for the GEJ are current standards. For the metastatic setting, biomarker profiling including Her2, PD-L1, MSS status is needed. Chemotherapy in combination with checkpoint inhibitors had improved the outcomes for patients with PD-L1 expression. Her2 positive patients should receive antiHer2 therapy added to chemotherapy. We describe the different evidences and recommendations based on the literature.

## Incidence and epidemiology

Gastric cancer (GC) incidence has been declining somewhat worldwide in recent decades. Nonetheless, it is the fifth most common cancer and the fifth leading cause of cancer deaths [1]. In Spain, the incidence is lower and GC represents the tenth most frequent tumor and the seventh cause of cancer mortality [2]. In contrast, gastro-esophageal junction cancer (GEJC) incidence is on the rise, especially in Western countries, probably due to changes in risk factors and more precise anatomic location registers. The median age at the time of diagnosis is 70 years; nevertheless, 15% are early onset GC and this subset is trending upward. Incidence differs by sex (men are 2–3 times more susceptible than women) and geographical location (incidence rates are higher in SE Asia, South America, and Eastern European countries).

The risk factors, background, and tumor biology differ based on tumor location: H pylori infection has been clearly correlated to distal or antral GC. These tumors follow a pattern of stepwise progression (known as the Correa Cascade [3], from normal mucosa to non-atrophic gastritis, atrophic gastritis with and without intestinal metaplasia (IM), dysplasia and, finally, to cancer. Smoking is a risk factor for which there is great evidence. Other risk factors with an intermediate level of evidence include diet (low in fruits and vegetables, excessive salt, high consumption of processed meats), atrophic gastritis, and autoimmune conditions.

- In GC involving the gastric fundus or body, alcohol and EBV infection have been correlated as risk factors.
- GEJC is associated with obesity and gastroesophageal reflux, the incidence of which is increasing in Western countries.

Some 10% of subjects with GC have a positive family history and > 3% are related to hereditary syndromes, the most common one being hereditary diffuse gastric cancer (HDGC) syndrome, caused by cadherin 1 gene (CDH1) alterations.

In terms of primary prevention, there is consistent evidence that eradication of H. pylori in healthy individuals and in patients with gastric atrophy, significantly lowers the future incidence of GC [4]. In high-risk East Asian countries, endoscopy-based screening programs have been implemented and resulted in higher detection rates of early-stage GC, with a substantial reduction in mortality.

## Diagnosis, staging, risk assessment and supportive care

Diagnosis should be made on the basis of a gastroscopic or surgical biopsy reviewed by an experienced pathologist, and histology should be reported according to the WHO criteria. Upper GI endoscopy is mandatory not only to describe the details of the anatomic location for surgical purposes, but also to be able to perform a direct biopsy. Diagnostic accuracy is 70% when made on the basis of a single biopsy and as high as 98% when several biopsies are taken. Multiple biopsies should be carried out to provide sufficient material for histological and molecular interpretation, particularly in the setting of ulcerated lesions. Approximately 90% of GC are adenocarcinomas (Ac), which are subdivided into diffuse and intestinal (Lauren classification) depending on histological appearance.

Thoracic and abdomino-pelvic CT scan is the preferred examination for staging and is highly accurate in detecting metastasis; albeit less sensitive for evaluating T and N spread. Echo-guided ultrasound (EUS) is more consistently accurate than CT for the diagnosis of malignant lymph nodes and is also indicated in very early stages to identify patients in whom mucosectomy can be offered.

The use of PET-CT scan in GEJC can improve staging by detecting the lymph nodes involved or metastatic disease. Furthermore, the diffuse Ac subtype, very common in GC, displays poor FDG uptake, and PET-CT has not been proven to improve N staging and reveals a 7% rate of false negative results [5].

Peritoneal metastases are present in almost 20% of GC at diagnosis and laparoscopy ± peritoneal lavage for malignant cells is recommended in all stage IB-III GC and in cT3-4 Siewert III GEJC, all of which are considered potentially resectable, to exclude radiologically occult peritoneal disease [6].

Surgery for GC entails high morbidity. Nutrition, body mass index (BMI), sarcopenia, hypercoagulation, comorbidities, age, and ECOG are predictive factors for mortality and outcomes in this setting. They are also predictive of overall survival (OS) in patients with advanced disease treated with systemic therapies. Therefore, they must be factored into the decision-making process prior to initiating any treatment strategy in all settings. A multidisciplinary evaluation, including a proper nutritional evaluation, inclusion of the patient into a prehabilitation programs, and geriatric assessment should be weighed based on each patient’s profile. Special efforts to assure nutritional intake must always be offered for all stages.

## Biomarkers

The acquisition of high-quality samples and coordination with anatomical pathology laboratories for processing is critical for biomarker/molecular assessments acquiring. The biomarkers currently under consideration include HER2 overexpression or amplification, mismatch repair (MMR) or microsatellite instability (MSI) determination, and PD-L1 Combined Positive Score (CPS). Using both the histological grade and subtype as defined by the Lauren classification is useful, since they provide insights as to prognosis and predict treatment response and, consequently, their inclusion in clinical assessments is recommended.

HER2 status is determined by immunohistochemistry (IHC) or in situ hybridization (ISH) techniques, applied to both biopsied material and surgical specimens [7]. A result that is commonly considered as positive is 3 + on IHC or 2 + on IHC + fluorescent in situ hybridization (FISH) positive. For endoscopic samples, a minimum of five tumor fragments is required, ideally between 6 and 8 fragments, to control for potential false negatives. HER2 positivity ranges from 5 to 25%, depending on location and subtype.

Approximately 8–25% of all patients exhibit high MSI or deficient mismatch repair (dMMR). To detect dMMR status, the first choice is IHC to determine the expression of the four genes involved in DNA MMR (MLH1, PMS2, MSH2, and MSH6) [I, A]. It is important to note that MLH1 and PMS2 are typically lost together, as are MSH2 and MSH6, although they can also be lost independently. In cases of uncertainty, confirmation via PCR to determine MSI-high status is recommended [8]. The presence of MSI is a marker for dMMR and is characterized by a hypermutated state of neoplastic cells; likewise, it is also important to contemplate referral to a geneticist for assessment if Lynch syndrome is suspected. These determinations should be performed at diagnosis to plan the therapeutic approach.

PD-L1 CPS by IHC is used to evaluate therapy with checkpoint inhibitors as first-line. CPS evaluates positivity in tumor cells, lymphocytes, and macrophages relative to the total number of viable tumor cells, and is expressed as a score. The cutoff points defined in clinical trials currently inform the clinical approach to be undertaken.

In the immediate future, the biomarker repertoire will include claudin 18.2 determination by IHC. This biomarker is a member of the claudin protein family, which consists of membrane proteins expressed in tight epithelial junctions. This protein, in particular, plays a role in maintaining cell-to-cell barriers in epithelial tissues. Clinical trial data have often used a positivity criterion of ≥ 2 + in at least 75% of cells, a scenario observed in 38% of the cases, and even higher (42.3%) in HER2 positive tumors. Such positivity is more prevalent among younger patients with a diffuse subtype. In claudin 18.2 positive tumors, CPS is ≥ 5 in 17%. This biomarker and anti-claudin antibodies are still awaiting approval.

Data from recent randomized controlled trials (RCT) suggest that other biomarkers, such as fibroblast growth factor receptor 2 (FGFR2) amplification (detected in 4–7% of cases) and FGFR2b overexpression (exhibited in 30% of the cases), might soon join the established biomarker panel. While EBV expression and tumor mutational burden (TMB) hold promise as indicators, more robust studies are warranted to confirm their clinical relevance. The usefulness of next-generation sequencing (NGS) in detecting neurotrophic tyrosine receptor kinase (NTRK) fusions remains dependent on the availability of effective therapies in a given region and is not yet deemed standard.

### ESCAT scores (9)

**Table:** 

Her 2	I, A
PD-L1 CPS	I, A
MSI	I, A
Claudine 18.2	II
FGFR2	II

## Treatment of resectable disease

Endoscopic resection (ER) is considered for tumors at a very early stage that are very unlikely suitable for in bloc resection. This can be performed at high-volume medical centers with extensive experience in these techniques. Endoscopic resection could be performed through different techniques. The European Society of Gastrointestinal Endoscopy (ESGE) recommends endoscopic submucosal dissection (ESD) as the recommended therapy for most superficial gastric neoplastic lesions. ESD is indicated for well differentiated Ac, clinically staged as intramucosal (T1a), of any size if not ulcerated, and ≤ 30 mm if ulceration is present. Endoscopic mucosal resection (EMR) is an alternative for lesions ≤ 10 mm with low likelihood of malignancy. [III, B] Additionally, ESGE guidelines suggest that gastric Ac ≤ 30 mm, submucosal (sm1), and well-differentiated, or ≤ 20 mm, intramucosal, and poorly differentiated, both with no ulcerative findings, can be considered for ESD, albeit this decision should be made on a case-by-case basis. [III, C] [10] A recent meta-analysis found that ER was correlated with fewer adverse events and shorter hospital stays compared to surgery, and, while ER was associated with lower complete resection rates and a higher risk of recurrence, the OS and 5-year cancer-specific survival were similar with both approaches [11]. Post-ER, strict monitoring with an endoscopic follow-up program is mandatory to detect recurrences that can be treated early, either by endoscopy or surgery.

### Surgery

Complete resection (ER) with negative margins is the treatment of choice for T1 tumors that do not meet criteria for endoscopic resection and for stage IB-III disease. [I, A] The type of resection, subtotal or total gastrectomy, and the extent of the margins depend on tumor location and histological subtype. The proper resection margin for ≥ T2 intestinal subtype tumors is considered to be 3 cm and 5 cm for those with diffuse histology. Routine splenectomy should not be performed in patients undergoing total gastrectomy for proximal gastric cancer, unless the spleen is involved or extensive hilar adenopathy is noted, as it increases operative morbidity without improving survival when compared to spleen-sparing surgery [I, A] [12]. There is consensus concerning lymphadenectomy including a minimum of 15 nodes for reliable staging. Fifteen years follow-up data from the Dutch D1D2 trial demonstrated a significant decrease in locoregional recurrence and lower gastric cancer-related mortality after the D2 procedure, while the long-term survival analysis of the Italian randomized clinical trial exhibited improved disease-specific survival and GC-related mortality in those patients with advanced disease and lymph node metastases who underwent D2 lymphadenectomy [13, 14]. Based on these studies, D2 lymphadenectomy is accepted as the standard of care in Western countries in teams with expertise and low postoperative morbidity. [I, A] Multimodal rehabilitation programs (ERAS, Enhanced Recovery After Surgery) include all aspects of optimal peri-operative care for patients undergoing gastrectomy. These measures have been shown to accelerate postoperative recovery, reduce the stress caused by surgery, and shorten hospital stay.

#### Recommendations

ER is an appropriate approach to treat very early GC in patients without suspected lymph node involvement and that meet the criteria for ER. [III, B].

The recommended approach for resectable GC is gastrectomy with D2 lymph node dissection performed by experienced surgeons in high-volume centers. [I, A].

### Complementary chemotherapy

Perioperative CT has been shown to significantly improve R0 rates, disease-free survival (DFS), and OS as opposed to surgery alone in two European, landmark phase III studies: the MAGIC trial [15], using six cycles (three before and three after surgery) of epirubicin-cisplatin-5-fluorouracil (ECF) and the FNCLCC/FFCD 9703 study, [16] that evaluated a perioperative cisplatin-5-fluorouracil regimen (Table 1). These results led to the adoption of perioperative CT as a standard approach for potentially resectable, clinical stage ≥ T2 GC or GEJ Ac, mainly in Europe and other Western countries. [I, A].

**Table 1:** Clinical trials of complementary therapy in gastroesophageal cancer [1]

Study	Study population	Treatment arms	Pathologic response	DFS	OS
Complementary chemotherapy					
MAGIC [15]	Lower esophageal (14.5%), GEJGEJ (11.5%) and gastric (74%) Ac ≥ cT2 and/or N + . N = 503	Surgery ECF × 3 → Surgery → ECF × 3	NR	NR NR HR = 0.66, p < 0.001	5-year OS: 23% vs. 36.3% HR = 0.75, p = 0.009
FFCD ACCORD-7 [16]	Resectable lower esophageal (11%), GEJ (64%), and gastric (23%) Ac. *N* = 224	Surgery CF × 2–3 → Surgery → CF × 3–4	NR	5-year DFS: 19% vs. 34%. HR = 0.65, *p* = 0.003	5-year OS: 24% vs. 38%. HR = 0.69, *p* = 0.02
AIO-FLOT4 [17]	GEJ (66%) and gastric (44%) Ac ≥ cT2 or N + . *N* = 716	ECF/ECX × 3 → Surgery → ECF/ECX × 3 FLOT × 4 → Surgery → FLOT × 4	pCR: 6% vs 16% (*p* = 0.02)	Median DFS: 18 m vs 30 m. HR = 0.75, *p* = 0.003	Median OS: 35 m vs 50 m. HR = 0.77, *p* = 0.012
PRODIGY [18]	Gastric (94.5%) and GEJ (4.5%) Ac T2-3N + /T4 any N. *N* = 266	D2 surgery → S-1 × 8 DOSx3 → D2 surgery → S-1 × 8	pCR 10.4% (*p* < 0.0001)	5-year PFS: 55.6% vs 60.4%. HR = 0.70, *p* = 0.023	3-year OS: 73.4% vs. 74.2%. HR = 0.84, *p* = 0.338
RESOLVE [19]	Gastric (63.5%) and GEJ (36.5%) Ac T4aN + or T4b any N. *N* = 1094	D2 surgery → CAPOX × 8 D2 surgery → SOX × 8 SOX × 3 → D2 surgery → SOX × 5	NR	3-year RFS: 51.5% vs 56.5% (HR = 0.77, *p* = 0.028) vs. 59.4% (HR = 0.86, *p* = 0.17)	3-year OS: NE vs NE (HR = 0.77, *p* = 0.045) vs. NE (HR = 0.81, *p* = 0.093)
ACTG-GC [21]	pStage II-III GC after D2 gastrectomy. N = 1059	D2 surgery D2 surgery → S-1 × 1 year	–	5-year RFS: 53.1% vs 65.4%. HR = 0.65, p < 0.05	5-year OS: 61.1% vs 71.7%. HR = 0.67, p = 0.003
CLASSIC [22]	pStage II-III GC after D2 gastrectomy. *N* = 1035	D2 surgery D2 surgery → CAPOX × 6 months	–	5-year DFS: 53% vs 68%. HR = 0.58, *p* < 0.0001	5-year OS: 61.1% vs 71.7%. HR = 0.67, *p* = 0.003
JACCRO GC-07 [23]	pStage III GC after D2 gastrectomy. *N* = 915	D2 surgery → S-1 × 1 year D2 surgery → S-1 + Docetaxel	–	3-year RFS: 57.4% vs 67.7%. HR = 0.715, *p* = 0.0008	3-year OS: 71.2% vs 77.7%. HR = 0.74, *p* = 0.0076
Complementary chemoradiotherapy					
CROSS [27]	Esophageal (76%) and GEJ (24%) SCC/Ac cT1N1 or cT2-3N0-1. *N* = 368	Surgery Neoadjuvant CT (wCP) + RT (41.4 Gy) → Surgery	pCR: 29%	Median DFS: 16.2 m vs 37.7 m HR = 0.64, *p* < 0.001	Median OS: 24 m vs 48.6 m HR = 0.70, *p* = 0.004
Neo-AEGIS [31]	Esophageal (68.8%) and GEJ (31.2%) Ac cT2-3N0-3 N = 377	EC(O)F(X) × 3/FLOT × 4 → Surgery → EC(O)F(X) × 3/FLOT × 4 CRT (CROSS) → Surgery	pCR: 4% vs 12% (*p* = 0.012)	Median DFS: 32.4 m vs 24 m HR = 0.89, *p* = 0.41	3-year OS: 55% vs 57%. HR = 1.03, *p* = 0.82
ESOPEC [28]	Esophageal and GEJ Ac cT1cN + cM0 and cT2-4acNxcM0	FLOT × 4 → Surgery → FLOT × 4 CRT (CROSS) → Surgery	pCR: 16.8% vs 10%	Median DFS: 38 m vs 16 m HR = 0.66; *p* = 0.001	Median OS: 66 m vs 39 m HR = 0.72; *p* = 0.023
Immunotherapy-based complementary treatment					
ATTRACTION-5 [34]	pStage III gastric (93%)/GEJ (7%) Ac after ≥ D2 gastrectomy. *N* = 755	CT (S-1 or CAPOX) + Placebo CT (S-1 or CAPOX) + Nivolumab	–	3-year RFS: 65.3% vs 68.4%. HR = 0.90, *p* = 0.4363	3-year OS: 78% vs 81.5%. HR = 0.88 (95% CI, 0.66–1.17)
VESTIGE [35]	ypN1-3 and/or R0-R1 gastric (56.5%)/GEJ (43.5%) Ac after preoperative CT and surgery. *N* = 240	Postoperative CT Ipilimumab + Nivolumab × 1 year	–	Median DFS: 23.2 m vs 11.93 m HR = 1.80, *p* = 0.0195	Median OS: NE vs. 23.13 m HR = 1.79, *p* = 0.0994
CheckMate-577 [36]	Stage II/III esophageal (58%) and GEJ (42%) SCC/Ac with residual pathologic disease after NA CRT → Surgery. *N* = 794	Placebo Nivolumab × 1 year	–	Median DFS: 10.4 m vs 22.4 m HR = 0.67 (95% CI, 0.55–0.81)	NR
DANTE [37]	Resectable gastric (38.5%)/GEJ (61.5%) Ac ≥ cT2 and/or N + *N* = 295	FLOTx4 → Surgery → FLOT × 4 FLOT + Atezo × 4 → Surgery → FLOT + Atezo × 4 → Atezo/3 w × 8	pCR: 15% vs 24%^a^	NR	NR
MATTERHORN [38]	Resectable gastric (67.5%)/GEJ (32.5%) Ac ≥ T2N0-3 or T0-4N + *N* = 948	FLOT × 4 → Surgery → FLOT × 4 FLOT + Durva × 4 → Surgery → FLOT + Durva × 4 → Durva/4 w × 10	pCR: 7% vs.19%. OR = 3.08, *p* < 0.00001	NR	NR
KEYNOTE-585 [39]	Resectable gastric (75.7%)/GEJ (20.5%) Ac ≥ cT3 and/or N + *N* = 1007	CT (XP/FP × 3 or FLOT × 4) → Surgery → CT CT + Pembro → Surgery → CT + Pembro → Pembro/3 w × 11	pCR: 2% vs 12.9% *p* < 0.0001	Median EFS: 25.3 m vs 44.4 m HR = 0.81, *p* = 0.0198^b^	Median OS: 58 m vs 60.7 m. HR = 0.90 (95% CI, 0.73–1.12)

*DFS* disease-free survival, *OS* overall survival, *RFS* relapse-free survival, *EFS* Event-free survival, *GEJ* Gastroesophageal junction, *Ac* Adenocarcinoma, *SCC* Squamous cell carcinoma, *NR* Not reported, *NE* Not evaluable, *HR* Hazard ratio, 95% *CI* 95% confidence interval, *pCR* Pathological complete response, *CT* Chemotherapy, *RT* Radiotherapy, *CRT* Chemoradiotherapy, *ECF* Epirubicin-cisplatin-5-fluorouracil, *ECX* Epirubicin-cisplatin-capecitabine, *CF* Cisplatin-5-fluorouracil, *FLOT* Docetaxel-oxaliplatin-leucovorin-5-fluorouracil, *DOS* Docetaxel-oxaliplatin-S-1, *SOX* Oxaliplatin-S-1, *CAPOX* Oxaliplatin-capecitabine, *wCP* Weekly carboplatin-paclitaxel

^a^All analyses are descriptive and explorative with no predefined hypothesis

^b^Threshold for significance was one-sided p = 0.0178

The German phase II–III AIO-FLOT4 study subsequently reported four cycles of pre- and post-operative FLOT (docetaxel, oxaliplatin, 5-fluorouracil, and leucovorin) to significantly prolong OS compared to the MAGIC schema (median of 50 vs. 35 months; HR = 0.77; *p* = 0.012) [17]. Based on these data, perioperative FLOT has been established as the standard of care for patients eligible for radical treatment. [I, A] For those unfit patients, other options considering a platinum- and fluoropyrimidine-based doublet or a modified dose of the FLOT schema can be considered. [II, B].

Moreover, two recent phase III trials have confirmed the superiority of neoadjuvant CT over D2 surgery followed by adjuvant CT in Asian patients with node positive or T4 gastric/GEJ cancer [18, 19], thereby providing evidence to support the use of this perioperative approach in East Asia, where adjuvant CT had historically been the complementary therapy of choice (Table 1). The benefit of the pre-operative approach is based on the potential chemotherapeutic effect in terms of downstaging and eradicating micrometastatic disease, along with better treatment tolerance (compared to the post-operative approach).

Adjuvant CT can be considered for those patients with stage ≥ IB GC who have undergone upfront potentially curative surgery, without prior neoadjuvant therapy (i.e., cases of uncontrollable bleeding and/or stenosis not amenable to palliative solutions), and who have had an adequate D2 lymphadenectomy with node-negative disease. [I, A] A meta-analysis published in 2010 [20] suggested a benefit in OS with this approach, which has been widely used in Asian countries. The ACTC-GC [21] and CLASSIC [22] phase III Asian studies have evidenced that 1 year of S1 or 6 months of CAPOX adjuvant CT, respectively, are associated with statistically significant DFS and OS benefit compared to observation following D2 gastrectomy of stage II-III GC. More recently, the addition of docetaxel to S1 has yielded better results than S1 alone in terms of 3-year relapse-free survival (RFS) (HR = 0.632; *p* < 0.001) for stage III disease [23] (Table 1). Based on these data, the recommended regimen when adjuvant CT is considered in Western populations is a doublet containing fluoropyrimidine + oxaliplatin or docetaxel for a total duration of 6 months.

Microsatellite instability (MSI) GC: post hoc analyses of prospectively conducted randomized trials have revealed the positive prognostic effect of MSI in surgically resected GC and the potential lack of benefit of perioperative or adjuvant CT in this population [24]. Although evidence is limited, treatment for MSI-high patients who have undergone curative surgery should be individualized. Dual immune checkpoint inhibition (ipilimumab + nivolumab in the NEONIPIGA and durvalumab + tremelimumab in the INFINITY study) [25, 26] has also proven to be promising as neoadjuvant treatment in cases with MSI-H tumors (pCR rate of 58.6–60%) and highlights the possibility of delaying or even avoiding surgery in highly selected subgroups (Table 1).

### Complementary chemoradiotherapy

Preoperative chemoradiotherapy (CRT) could be considered as an acceptable therapeutic approach for patients with GEJ adenocarcinoma over surgery alone. The phase III CROSS trial demonstrated a significant survival benefit with preoperative CRT (weekly carboplatin plus paclitaxel, and 41.4 Gy for 5 weeks) compared to surgery alone. Nevertheless, this study mixed different tumor types and the survival benefit was higher for squamous cell carcinoma of esophagus, GEJ adenocarcinoma patients also obtained recurrence-free and OS benefit [27] [Level I, Grade A]. However, new data regarding the benefit of perioperative chemotherapy (CT) vs preoperative CRT for GEJ adenocarcinoma was recently presented at ASCO annual meeting 2024 according to findings from the phase 3 ESOPEC trial (NCT02509286) favoring the FLOT strategy [28]. ESOPEC was a prospective multicenter study that enrolled patients with esophageal adenocarcinoma across 25 sites in Germany. Eligible patients needed to be at least 18 years old, have received no prior abdominal or thoracic radiotherapy, have an ECOG performance status of 2 or less, and have adequate organ function. Patients were also required to have pretreatment stage cT1N + , M0 or cT2-4a, N0/ + , M0 disease. Patients were randomly assigned 1:1 to receive either the FLOT or CROSS protocol. Those in the FLOT arm received repeated doses of 5-fluorouracil, leucovorin, oxaliplatin, and docetaxel every 2 weeks over 4 neoadjuvant cycles prior to surgery and 4 adjuvant cycles after surgery. In the CROSS arm, patients received neoadjuvant radiation therapy and concurrent chemotherapy with carboplatin and paclitaxel 5 weeks prior to surgery. The primary end point was OS. Secondary end points included progression-free survival, recurrence-free survival, and postsurgical quality of life. Both arms were well balanced. The results showed, at a median follow-up of 55 months, patients who received the FLOT protocol (*n* = 221) achieved a median OS of 66 months compared with 37 months among those who were treated with the CROSS protocol (*n* = 217). The 3-year OS rates were 57% vs 51%, respectively and a median PFS of 38 vs 16 months respectively. Pathological completed remission was seen in 16.8 and 10% respectively. It’s important to note that only 67.7% of the patients included in the CROSS arm were able to complete the neoadjuvant strategy while 87.3 and 52.5% completed the pre-operative and post-operative FLOT respectively,

Previous evidence included the meta-analysis by Petrelli showed that preoperative CRT did not significantly benefit in the risk of distant metastasis nor OS, although may have a positive impact in the risk of relapse, the risk of locoregional failure and higher pathological response [29] and the data from the phase III Neo-AEGIS trial compared the administration of preoperative CRT (CROSS) with perioperative CT (MAGIC/FLOT) in patients with adenocarcinoma of the esophagus or GEJ. OS was non-inferior for perioperative CT (3y-OS 55%) compared with preoperative CT (3y-OS 57% and HR 1.01). Nevertheless, the majority of patients were treated with the old MAGIC schema, which is currently obsolete [30, 31] [Level I, Grade C] The question that stills open is if perioperative FLOT is better than the multimodal approach for high risk patients who received CRT followed by surgery, and adjuvant immunotherapy based on the data of the Checkmate 577 discussed in the next section.

Postoperative chemoradiotherapy (CRT) has demonstrated survival benefit in patients with gastric and GEJ cancer over surgery alone. The old phase III INT-0116 trial reported a statistically significant benefit for both RFS (HR 1.51; *p* < 0.001) an OS (HR 1.32; *p* = 0.0046) with postoperative 5FU-based CRT compared to the observation [32]. Concerns about the credibility of this study are reasonable considering that about 90% of patients had underwent to insufficient lymphadenectomies. Data from the Nordic CRITICS trial confirms the lack of benefit on post-operative radiotherapy, and approach that should be individualized only in those patients that had infratherapeutic lymphadenectomies (III, B) and R1 resections (III,C) [33]. Results of the TOP-GEAR, and CRITICS-II (two randomized trials that are currently exploring the role of preopertaive chemo-radiotherapy in GC) are still pending.

### Complementary immunotherapy

Two large, randomized studies examining the potential role of adjuvant immunotherapy in resectable esophagogastric Ac have yielded negative results (Table 1). The ATTRACTION-5 study, a phase III trial comparing adjuvant CT + nivolumab to placebo + nivolumab in Asian patients with pathological stage III gastric/GEJ cancer, failed to meet the primary endpoint of RFS [34]. Similarly, the VESTIGE phase II study indicated that adjuvant nivolumab/ipilimumab did not improve DFS compared to adjuvant CT in European patients with gastroesophageal Ac at high risk for recurrence (N + or R1) following CT + surgery [35]. Interestingly, the VESTIGE trial confirmed the benefit of performing post-operative CT in all patients irrespective of the pathological response to pre-operative CT. In contrast to these results, the phase III CheckMate 577 trial has reported improved DFS with the addition of one year of nivolumab vs. placebo in a mixed pool of patients that aggregated squamous cell carcinoma and esophageal and GEJ Ac who had evidence of residual pathological disease following neoadjuvant CRT and surgery (11 versus 22.4 months; HR = 0.69; *p *< 0.001) [36]. This results allowed the approval from EMA for this indication and it´s funded by the Spanish health system. Consequently, adjuvant nivolumab should only be prescribed in GEJC patients who have been treated with CRT followed by surgery, regardless of PD-L1 expression status. [I, A] The overall survival results are still pending.

In the peri-operative setting, the results from phase II and III trials incorporating immunotherapy to perioperative CT in resectable gastric/ GEJ cancer have exhibited statistically significant improvements in pathological complete response rates and pTNM compared with chemo alone [37–39]. The Keynote 585 study evaluated the addition of pembrolizumab to CT in the perioperative setting. The results of event-free survival (EFS) data have recently been reported. EFS was longer in the experimental arm (44.4 versus 25.3 months; HR 0.81 [95% CI, 0.67–0.99]; *p* = 0.0198); regardless, these results failed to achieve statistical significance. In consequence, no studies have yet evinced improvement as far as survival parameters are concerned at the moment this guideline is published.

### Other complementary systemic treatments

The introduction of targeted therapies with proven clinical activity in resectable metastatic GC has paved the way to the concept of personalizing treatments in this setting.

Regarding HER2-targeted approaches in HER2-positive early GC, trastuzumab in combination with perioperative FLOT has been assessed in the phase II HER-FLOT study [40], which reached its primary endpoint (pCR > 20%), achieving a pCR rate of 21.4%. The phase II-III AIO-PETRARCA study [41] explored the role of dual HER2 inhibition with trastuzumab and pertuzumab in combination with FLOT compared to CT alone. The primary endpoint of the phase II portion of the trial was the pCR rate, which was achieved in 35% of the patients in the experimental arm vs. 12% in the control group (*p* = 0.019), albeit at the expense of increased toxicity. Unfortunately, and due to the sponsor's decision, this study could not continue to phase III, which precluded evaluation of this strategy in terms of survival. In contrast to these results, the INNOVATION phase II study [42] tested the inclusion of trastuzumab alone or with pertuzumab into perioperative CT. The trial failed to reach the primary endpoint of demonstrating the superiority of the double blockade, probably attributable to unacceptable toxicity. Interestingly, the addition of trastuzumab alone to the perioperative CT schedule proved to be effective. Follow-up data including survival are necessary to define the clinical value of this regimen.

In relation to VEGF inhibitors, phase II studies have explored the activity of bevacizumab or ramucirumab in addition to perioperative CT, failing to improve the outcomes.

Other pathways involving claudin18 isoform 2 (CLDN18.2), FGFR, and DNA damage repair proteins may be of interest in certain patients, but no trials in early-stage disease are available yet during this guideline development.

### Follow-up, long-term implications, and survivorship

There are no randomized trials that address the survival benefit of surveillance strategies for resected gastric cancer patients. In a recent meta-analysis of 15 observational studies, planned surveillance did not achieve significantly better detection of recurrence or OS benefit for gastric cancer patients. Despite this, a follow-up suggestion is shown in Table 2. [Level III, Grade C].

**Table 2:** Proposed surveillance protocol for resected gastric cancer patients

Procedure	Frequency
History and physical examination	Every 6 months for 3 years Every 12 months for 2 years
Laboratory test (blood counts, chemistry profile, iron, and vitamin B12)	Every 6 months for 3 years Every 12 months for 2 years
CT scan	Every 6 months for 3 years Every 12 months for 2 years
Endoscopy	As clinically indicated
Other procedures	As clinically indicated

Finally, gastric cancer patients should be continually monitored for nutritional status, with a special emphasis on iron and vitamin B12.

## Treatment of advanced disease

Systemic treatment for advanced GC, including locally advanced unresectable and metastatic disease, includes CT, antiHER2 therapy, immunotherapy, and other targeted therapies.

### First-line chemotherapy

Early CT trials in advanced GC demonstrated how CT improved survival compared to best supportive care and later proved the benefit of CT combinations over single agent treatment. [43]. Table 3 summarize the magnitude of benefit from the different strategies that supports our clinical practice in the perioperative and advanced setting according to ESMO magnitude of clinical benefit scale (https://www.esmo.org/guidelines/esmo-mcbs)

The current standard first-line CT combinations for advanced GC include a platinum compound and fluoropyrimidine doublet. The first backbone to be established was the combination of cisplatin and 5-fluorouracil administered by continuous infusion (5-FU c.i.), [43] while subsequent phase III clinical trials incorporated oxaliplatin and capecitabine as alternative compounds to cisplatin and 5-FU c.i., respectively [44, 45]. For older or frail patients, oxaliplatin choice as well as the use of reduced doses of oxaliplatin-based CT combinations have exhibited better safety profile and, at least, comparable survival outcomes versus cisplatin and standard dosing, respectively [44, 46].

Triplet combination CT in advanced GC includes the addition of docetaxel or anthracycline to the platinum and fluoropyrimidine doublet, although at the expense of increased toxicity [47, 48].

### First-line HER2-targeted therapies

HER2 overexpression or amplification has been identified as a potential prognostic factor and is considered a valid therapeutic target. In the ToGA trial, the addition of the HER2-targeted monoclonal antibody trastuzumab to cisplatin and fluoropyrimidine (capecitabine or 5-FU c.i.) doublet CT significantly improved OS with low and manageable additional toxicity in patients with previously untreated HER2 positive locally advanced or metastatic GC or GEJ cancer compared to CT alone, specifically with increased efficacy in patients with tumors that were immunohistochemistry (IHC) 3 + regardless of fluorescence in-situ hybridization (FISH) status, or that were IHC 2 + and FISH positive [49]. Thenceforth, trastuzumab plus a platinum-based drug and fluoropyrimidine have been regarded as the first-line standard of care treatment in patients with HER2 positive (IHC 3 + or IHC 2 + and FISH positive) advanced GC.

Further attempts to increase antiHER2 therapy efficacy by adding pertuzumab, a humanized monoclonal HER2-targeted antibody that binds to a different epitope on the HER2 receptor protein than trastuzumab, to trastuzumab plus a platinum-based drug and fluoropyrimidine failed to demonstrate significant improvement in OS in the phase III JACOB trial. [50].

On the other hand, the recently reported results of the phase III KEYNOTE-811 study revealed that the addition of pembrolizumab to the TOGA schema improved survival outcomes for those patients with PDL1 CPS ≥ 1. [I, A] This therapy strategy was approved by EMA for patients Her2 positive with PDL1 CPS ≥ 1 but is not funded by the Spanish government yet.

### First-line immunotherapy for HER 2 negative

The use of immunotherapy as first-line treatment in combination with chemotherapy for gastric and GEJ Ad is guided by the PD-L1 expression status assessed by the CPS score.

The first positive study supporting this strategy is based on the Phase III Checkmate 649 study, which evaluated the addition of nivolumab to an oxaliplatin and fluoropyrimidine doublet (capecitabine or 5-fluorouracil). The primary endpoint was the OS and PFS for patients with PD-L1 expression CPS ≥ 5. The results showed after a 24-month follow up, substantiated OS benefit (HR 0.70, 95% CI 0.61–0.81, *p* < 0.0001) and PFS (HR 0.70, 95% CI 0.61–0.81, *p* < 0.0001) with respect to CT. This strategy was approved by EMA for this subgroup, and is a treatment strategy funded by the Spanish government. As a secondary endpoint, the OS and PFS was also tested for all randomized showing also significant improvement with the incorporation of immunotherapy to the strategy. (HR 0.70, 95% CI 0.71–0.88) and progression-free survival (PFS) (HR 0.79, 95% CI 0.70–0.89) respectively. The results from 48 months of follow-up continue to support these results. [51] The study included also an arm testing the combination of nivolumab and ipilumumab vs. CT which in this context did not achieve statistical significance [52, 53].

A second important study that supports the use of immunotherapy added to CT in the first line strategy for GC is the Keynote-859 trial which compared the addition of pembrolizumab to a platin + fluoropyrimidine-based CT doublet vs CT. The primary endpoint was the OS benefit, and secondary endpoints included the PFS, DOR, ORR and safety and with a significant improvement for the OS (HR 0.78, 95% CI 0.70–0.8, *p* < 0.0001) and PFS (HR 0.76, 95% CI 0.67–0.85, *p* < 0.0001) benefit with respect to both variables in the overall study population; the magnitude was greater in the CPS ≥ 1 subgroup (HR 0.73, 95% CI 0.64–0.83) and for PFS (HR 0.72, 95% CI 0.63–0.82), as well as in the CPS ≥ 10 group (OS: HR 0.64, 95% CI 0.52–0.77; PFS: HR 0.62, 95% CI 0.51–0.75) [54]. Based on this benefit, this strategy got the EMA approval for patients with PD-L1 CPS ≥ 1 subgroup. (Not funded by the Spanish government yet).

On the other hand, the Keynote 590 study evaluated in patients with adenocarcinoma and squamous cancer of the esophagus and esophagogastric junction (EGJ) the role of adding pembrolizumab to cisplatin and fluorouracil, with the primary objectives being overall survival (OS) in patients with squamous cell carcinoma (SCC) PD-L1 CPS ≥ 10; OS and PFS in patients with SCC; in all patients with PD-L1 CPS ≥ 10 and in all patients of the study, showing a benefit in OS and PFS for all primary objectives of the study, with better benefits for the population with CPS ≥ 10, therefore achieving approval of the EMA for this subgroup and it is a treatment that is funded by the Spanish national health system [55].

Similarly, positive phase III studies have been reported when other anti-PD-1 agents, such as sintilimab (ORIENT-16) or tislelizumab (RATIONAL 305), have been added, although they have yet to be approved by the EMA.

The presence of MSI-H or dMMR is highly important to define a subgroup who deeply benefit from immunotherapy.

The participants with MSI-H or dMMR, displayed an OS benefit in the Checkmate 649 trial when nivolumab was added in 44 patients (median OS 38.7 months vs 12.3 months, HR 0.38, 95% CI 0.17–0.84) [52, 53].

Likewise, a meta-analysis of first-line and successive lines of therapy evidenced benefit in response rate (RR) when pembrolizumab was used, regardless of the treatment line in the subgroup of patients with MSI-H or cMMR [56].

#### Recommendations

The addition of nivolumab to an oxaliplatin and fluorouracil-based CT doublet to treat gastric and GEJ Ac is recommended in patients with a CPS ≥ 5 [I, A].

Incorporating pembrolizumab to a platin and fluorouracil doublet is recommended in subjects with gastric and GEJ Ac and a CPS ≥ 1 [II, A], albeit the benefit is greatest in the CPS ≥ 10 subgroup [I, A].

The addition of pembrolizumab in monotherapy or the combination of CT and nivolumab can be contemplated in individuals with MSI-H or dMMR [II, B].

### Other new therapeutics in first-line

Anti-Claudin 18.2 therapy is expected to be integrated into the therapeutic armamentarium before long. [I, A] The phase III Spotlight RCT compared FOLFOX plus zolbetuximab or placebo in tumors with moderate-to-strong claudin expression (in > 75% of tumor cells) [57]. The primary endpoint was PFS. The trial pointed toward improved PFS from 8.67 to 10.6 months (HR 0.75, 95% CI 0.58–0.94, p = 0.006). Moreover, median OS rose from 15.5 months to 18.2 months (HR 0.75, 95% CI 0.60–0.93, *p* = 0.0053).

Meanwhile, the phase III GLOW RCT largely confirmed these findings. The eligibility criteria and design mirrored the Spotlight trial, except for the use of CAPOX as the CT backbone, in combination with either zolbetuximab or placebo [58]. The experimental group exhibited improved PFS with 8.2 months as compared to 6.8 months in the control arm (HR 0.68, 95% CI 0.54–0.86, *p* = 0.0007). Median OS also rose from 12.1 to 14.3 months (HR 0.77, 95% CI 0.61–0.96, *p* = 0.01).

Furthermore, the anti-claudin strategy is amenable to alternative approaches. Notably, humanized claudin18.2-redirected CAR-T cells or anti-claudin 18.2 antibody drug conjugates (ADC) are promising strategies currently in the early phases of research. [III, C] [59].

Awaiting validation, targeted anti-FGFR therapy is another approach likely to be adopted in the coming years. [II, B] As of now, in the phase II FIGHT trial that included individuals exhibiting FGFR2b overexpression or FGFR2 amplification were randomized to receive FOLFOX ± bemarituzumab. In the experimental arm, median PFS improved from 7.4 to 9.5 months (HR 0.68, 95% CI 0.44–1.04). Meanwhile, OS increased from 12.9 months to not yet reached (HR 0.58, 95% CI 0.35–0.95) [60]. Should the phase III FORTITUDE 101 and 102 trials confirm a favorable risk–benefit profile, this strategy could expand the therapeutic arsenal.

Other targeted therapies contingent on availability, such as NTRK inhibitors for fusions (e.g., entrectinib, larotrectinib), may be of interest in certain cases, subject to individual assessment. [II, B].

#### Role of surgery in limited metastatic disease

Recently the results of the phase III IKF-575/RENAISSANCE have been communicated, This trial randomized 139 pts with limited-metastatic adenocarcinoma of the stomach or esophagogastric junction to be treated with chemotherapy/targeted therapy alone vs. chemotherapy/targeted therapy followed by radical surgical resection. The primary endpoint was overall survival and was not met due to increased early mortality in the surgery arm Pts with RPLN metastases only seemed to benefit most from the surgical approach (mOS, 30 vs.17 months; 5y OS 38% vs. 19%; still having increased early mortality), while pts showing no response to chemo (mOS, 13 vs. 22 months) or pts with peritoneal disease (mOS, 12 vs. 19 months) derived a detrimental effect [61].

#### Second and subsequent lines

Up to 50% of GC and GEJC patients are fit enough to receive a second line of treatment, fewer in the case of subsequent lines. The benefit of a second CT line has been widely demonstrated in terms of survival and quality of life. Standard options are paclitaxel, docetaxel, and irinotecan with equivalent efficacy, yet different toxicity profiles. [I, B] The addition of the anti-vascular endothelial growth factor receptor 2 antibody ramucirumab to paclitaxel improves effectiveness, according to the phase III RAINBOW trial [62] [I, A].

When considering immunotherapy, the phase II KEYNOTE-158 basket trial proved the efficacy of pembrolizumab in previously treated advanced patients with dMMR/MSI-H tumors. For GC patients, ORR was 45.8% and median PFS was 11 months, with median OS not yet reached [63]. Considering that such results have never been previously reported, pembrolizumab is the preferred treatment option in this setting. [II, A].

Upon progression to trastuzumab-based line of therapy, HER2-positive patients should be considered separately. While earlier trials with the combination of trastuzumab + lapatinib and with trastuzumab emtastine had been negative, the paradigm of these patients has changed with the incorporation of the ADC trastuzumab deruxtecan (T-Dxd). The randomized phase II trial conducted in Asia evinced the advantage of T-Dxd over CT [64]. These results were confirmed in a Western population, in a single arm, phase II trial [65] that evinced that T-Dxd yielded an ORR of 42%, median PFS of 5.6 months, and median OS of 12.1 months in HER2-positive GC and GEJC patients that had progressed to first-line trastuzumab-based treatment. [II, B]. The results of this last study made it possible for EMA approval for trastuzumab-deruxtecan following post-trastuzumab progression. Results of the current global phase III DESTINY-Gastric04 trial will establish the actual benefit of this drug in this setting.

In the third-line setting, oral treatment with trifluridine-tipiracil has the strongest evidence, with proven efficacy for survival and time to ECOG/PS deterioration based on the phase III TAGS trial. [66] [I, A]. Nevertheless, this treatment is currently not reimbursed in Spain. Other options include a taxane or irinotecan, depending on the second line therapy [II, A].

**Fig. 1:**
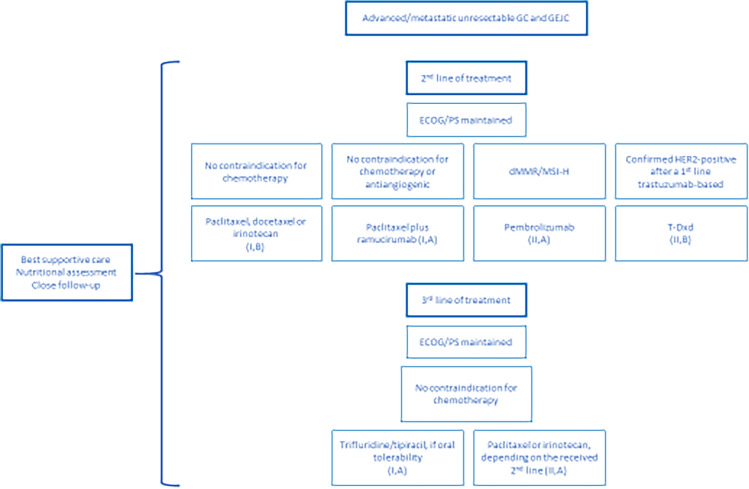
Proposed algorithm for second and subsequent lines of treatment in advanced GC

An algorithm proposed for second and subsequent lines is presented in Fig. 1. Special consideration must be paid to the need for close symptom monitoring and nutritional assessment during all GC and GEJC treatment lines. Close follow-up radiological imaging will early detect tumor progression and prevent potential treatment-related toxicities.

**Table 3:** ESMO- MCBS scores

Study	Drug	Setting	OS gain	EMBCS score	HR	Year
FLOT	Oxaliplatin/ 5-FU-Docetaxel	perioperative GC + UEG	15 months (median) 5-years: 9%	A	0.77 (0.63–0.94)	2019
CM 577	Nivolumab	adjuvant UEG	DFS gain 11.4 months	A	0.69 (96.4%CI 0.56–0.86)	2021
CM 649	Nivolumab + doublet platinum/ fluoropyrimidine CT	First line metastatic GC, UEG PD-L1 CPS ≥ 5	3.3 months	4	0.70 (0.61–0.81)	2021/ updated 2023
KN 590	Pembrolizumab + doublet platinum/ fluoropyrimidine CT	First line metastatic UEG PD-L1 CPS ≥ 10	4.1 months	4	0.62 (0.49–0.78)	2021
KN 859	Pembrolizumab + doublet platinum/ fluoropyrimidine CT	First line metastatic GC,UEG PD-L1 CPS ≥ 1	1.6 months 2 year 12%	4	0.74 (0.65–0.84)	2023
KN 811	Pembrolizumab + trastuzumab + doublet platinum/ fluoropyrimidine CT	First line metastatic Her2 positive GC, UEG CPS ≥ 1	4.3 months at 38 months follow-up	2	0.81 (0.67–0.98)	2022/ 2023
KN158	pembrolizumab	Second line metastatic MSI GC	ORR 29% DOR 9 months, median not reached	3		2022
Destiny 02	Trastuzumab-deruxtecan	metastatic setting after trastuzumab progression GC, UEG	ORR 42% DOR 9 months	2		2023
